# Is Hypercalcemia a Frequent Complication following Local Use of Calcium Sulfate with Antibiotics for the Treatment of Extremity Posttraumatic Osteomyelitis? A Preliminary Study

**DOI:** 10.1155/2019/7315486

**Published:** 2019-03-31

**Authors:** Nan Jiang, Guan-qiao Liu, Jia-jun Yang, Qing-rong Lin, Yan-jun Hu, Bin Yu

**Affiliations:** ^1^Department of Orthopaedics & Traumatology, Nanfang Hospital, Southern Medical University, Guangzhou 510515, China; ^2^Guangdong Provincial Key Laboratory of Bone & Cartilage Regenerative Medicine, Southern Medical University, Guangzhou 510515, China

## Abstract

**Background:**

Previous study had reported hypercalcemia as a frequent complication (20%) following local use of antibiotic-eluting calcium sulfate (CS) during treatment of periprosthetic joint infections (PJIs). However, whether this complication may occur in patients who receive local CS implantation for management of posttraumatic osteomyelitis (OM) remains unclear.

**Methods:**

Between April 2016 and May 2017, we included 55 patients with extremity posttraumatic OM who received local antibiotic-loaded CS therapy. Serum calcium levels were detected preoperatively and on the 1st, 3rd, and 7th postoperative days (PODs). Comparisons were performed regarding serum calcium levels among the four time points and between two different CS volume groups (≤ 20 cc group and > 20 cc group). Additionally, potential associations were examined regarding CS volume and preoperative calcium level with postoperative calcium levels, respectively.

**Results:**

Altogether 46 males and 9 females were included, with a median CS volume of 20 cc. Outcomes showed that prevalence of asymptomatic hypocalcemia was more frequent, with 16.4% before surgery and 60%, 53.8%, and 25% on the 1st, 3rd, and 7th PODs, respectively. Hypercalcemia was not found in any patients, at any time point. In addition, significant differences were identified regarding serum calcium levels among different time points, suggesting significantly decreased calcium levels on the 1st (*P *< 0.001) and 3rd PODs (*P *< 0.001) and back to near preoperative level on the 7th POD (*P *= 0.334). However, no statistical differences were observed regarding serum calcium levels between the two CS volume groups at any time points (*P *> 0.05). Moreover, no significant links were identified between CS volume and postoperative calcium levels (*P *> 0.05). Serum calcium levels on the 3rd (*P *= 0.019) and 7th PODs (*P *= 0.036) were significantly associated with the preoperative calcium level.

**Conclusions:**

In contrast to what had occurred in PJI patients, asymptomatic hypocalcemia appeared to be more frequent in this cohort with posttraumatic OM. Hypercalcemia may be an infrequent complication before and after local CS use for the treatment of extremity posttraumatic OM.

## 1. Introduction

As one of the most frequent types of extremity chronic osteomyelitis (COM) [1], posttraumatic OM usually arises as a sequelae or complication following open fractures or orthopaedic surgery. Currently, this disease still represents great challenges to clinicians because of its long disease course and a higher rate of infection recurrence, with higher risks of physical and psychological disabilities. Treatment algorithms consist of radical debridement, systematic combined with local antibiotics, and, if necessary, strategies for bone defect reconstruction [2]. Of these, delivery vehicles of local antibiotics act as an important role in the treatment of posttraumatic OM. Although polymethylmethacrylate (PMMA) remains to be one of most frequently used antibiotic carriers in the present, it has several drawbacks, such as being nonbiodegradable and not applicable for heat-sensitive antibiotics, with highly variable elution kinetics [3].

Recently, calcium sulfate (CS) is increasingly used as a novel antibiotic vehicle for the management of osteoarticular infections and has achieved satisfying clinical efficacy [4–7]. Compared with PMMA, CS has many advantages, such as being completely biodegradable by gradual dissolution, carrying a wider range of antibiotics and stimulating new bone formation [5]. However, everything contains two sides. The above-mentioned superiorities of CS never mean it has no flaws. One of the most frequent complications following CS local use is aseptic wound drainage [8]. Other uncommon adverse reactions during treatment of bone and joint infections include infection recurrence, fracture risk, and heterotopic ossification (HO).

In a recent study [9], Kallala and Haddad reported that 3 out of 15 patients (20%), who received CS beads for the treatment of periprosthetic joint infection (PJI), experienced postoperative hypercalcemia. One patient (33.3%) developed clinical symptoms and received further therapy. The potential risk of hypercalcemia following local CS use has aroused wide attention regarding its safety for clinical application. Although hypercalcemia is listed as a definite contraindication in the instruction of CS production [9], up till now, the complication of hypercalcemia remains seldom reported.

In our department, CS is selected as a local antibiotic carrier for the treatment of extremity posttraumatic OM. The already reported complication alerted us whether hypercalcemia may also occur in OM patients who received local CS implantation. Therefore, we performed the present study in patients with extremity posttraumatic OM to investigate (1) prevalence of hypercalcemia and hypocalcemia before and after CS implantation, (2) changes of serum calcium levels by time and differences of serum calcium levels by CS volume, and (3) potential links between CS volume and postoperative serum calcium levels, as well as between preoperative serum calcium level and postoperative serum calcium levels.

## 2. Patients and Methods

### 2.1. Study Design, Setting, and Data Source

This observational study was performed in a tertiary medical center in Southern China. Patient data were collected from those who were diagnosed as having posttraumatic OM and received local CS therapy between April 2016 and May 2017. Informed consent was obtained from all individual participants or legal guardian included in the study. The study protocol was approved by medical ethical committee of the hospital.

### 2.2. Definition, Sample Collection, and Inclusion and Exclusion Criteria

Posttraumatic OM is defined as a long term (over 10 weeks [10]) bone with or without soft tissue infection, which occurs as sequelae or a complication following open fractures or orthopaedic surgery. Diagnosis of posttraumatic OM is built on any of the following three points: (1) histopathological test of the intraoperative specimens confirms infection; (2) cultures from at least two suspected infection sites reveal the same pathogen; (3) a definite sinus tract/fistula connects directly the bone or the implant.

Peripheral blood samples were collected from cubital veins of the fasting patients with a tourniquet in the middle of the upper arm. The whole procedure of sample collection took approximately 30 seconds. Then, calcium level was detected and normal range of calcium level provided by the Medical Clinical Laboratory of our hospital is between 2.20 and 2.65 mmol/L. Therefore, hypocalcemia means serum calcium level is lower than 2.20 mmol/L, with hypercalcemia of over 2.65 mmol/L.

Eligible patients in the present study were those with definite diagnosis of extremity posttraumatic OM and received surgical interventions including local CS implantation. Additionally, eligible patients should have normal renal and parathyroid functions. Excluded from the present study were patients with acute osteomyelitis or joint arthritis, non-extremity bone infection, and hematogenous or diabetic foot (DF) OM, without local CS use. In addition, patients with comorbidities (e.g., malignant tumors) that may affect serum calcium level or listed as contraindications of CS product (Stimulan Rapid Cure; Biocomposites, Ltd., Staffordshire, UK) were also excluded.

### 2.3. Surgical Procedures

Surgical procedures were performed according to Cierny-Mader classifications [11]. According to production instruction, 1 g of vancomycin or 2 ml of gentamicin per 10 cc CS was mixed using the solvent provided by the manufacturer. For type I of intramedullary infected patients, Reamer-Irrigator-Aspirator (RIA) system [12] was performed initially. Then, gel-like CS or CS beads with vancomycin were placed into the medullary cavity. For type II (superficial), type III (localized), and type IV (diffused) infected patients, CS (beads or block-shaped or both) was placed or filled in the defected site following radical debridement for bone and soft tissues. For calcaneal OM, CS was placed in the defected site following “eggshell-like” style of debridement, which was described previously [13].

### 2.4. Statistical Analysis

Statistical analysis was conducted by the Statistical Product and Service Solutions (SPSS) 17.0 software (SPSS Inc., Chicago, IL, USA). Data distributions were evaluated for normality using the Kolmogorov-Smirnov test. Continuous variables were expressed as mean ± standard deviation or median with interquartile range (IQR) depending on data distribution. For normally distributed data, Student's t-test or one-way analysis of variance (ANOVA) was used to compare the differences between two independent or more than two groups. Otherwise, Mann–Whitney U test or Kruskal-Wallis H test was used. Bonferroni or Dunnett's T3 method was used for post hoc multiple comparisons following ANOVA test based on the outcomes of homogeneity test of variance. Dichotomous variables were expressed as percentages and events with totals. Chi-square test was used to compare differences of rates among different groups. Spearman and Pearson correlations were applied to investigate potential links between CS volume and postoperative serum calcium levels and potential associations between preoperative serum calcium level and postoperative serum calcium levels, respectively. Statistically significant difference was defined as* P* value of ≤ 0.05.

## 3. Results

### 3.1. Patient Demographics

A total of 55 patients (46 males and 9 females) were included. The mean age was 40.2 ± 15.1 years (range 3 to 76 years). The median CS volume was 20 cc, IQR (10 cc, 30 cc). The mean length of bone defect of the long bones (available in 35 patients) was 6.7 ± 2.9 cm (range 1.2 to 16.2 cm).

### 3.2. Characteristics of Posttraumatic OM

#### 3.2.1. Infection Side and Site

There were 22 cases and 31 cases with infection on the left and right body side, with 2 cases on bilateral sides. Single site infection was found in 51 patients, with tibia (32 cases, 62.7%) as the most frequent site, followed by calcaneus (8 cases, 15.7%) and femur (7 cases, 13.7%), respectively (Table 1).

#### 3.2.2. Cierny-Mader Classification

The most common anatomy classification was type IV (38 cases), followed by type III (8 cases), type I (5 cases), and type II (4 cases), respectively. With regard to host classification, forty-two patients were classified as type A, with 13 cases as type B.

#### 3.2.3. Pathogen Culture Outcome

Altogether 45 patients received pathogen culture, with a positive culture rate of 57.8% (26 cases). Among the 24 patients with monomicrobial associated infection, the most frequently detected pathogen was* Staphylococcus aureus* (6 cases, 25%), followed by* Pseudomonas aeruginosa *(3 cases, 12.5%) and* Enterococcus faecalis *(3 cases, 12.5%), respectively (Table 1).

### 3.3. Prevalence of Hypercalcemia and Hypocalcemia before and after Surgery

**Figure 1:**
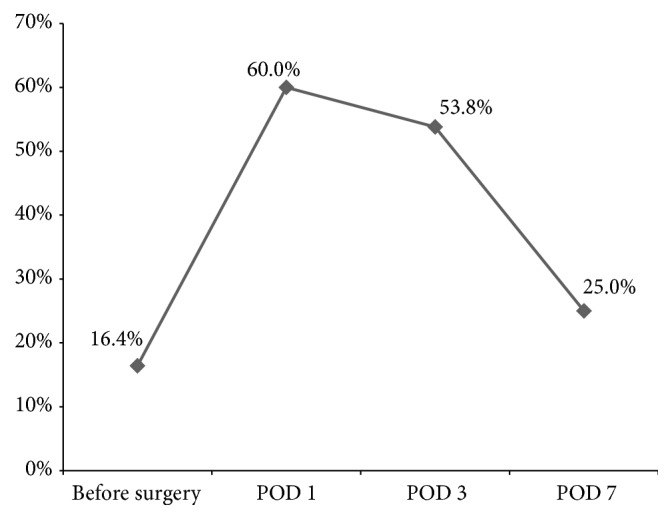
Changes of hypocalcemia prevalence before and after CS implantation surgery.

Prevalence of hypocalcemia was more frequent, which achieved 16.4% (9/55) preoperatively, with 60% (33/55), 53.8% (28/52), and 25% (8/32) on the 1st, 3rd, and 7th postoperative days (PODs), respectively (Figure 1). Nonetheless, no patient experienced clinical symptoms of hypocalcemia.

With respect to effect of CS volume on the prevalence of hypocalcemia, outcomes revealed that no significant differences were identified regarding percentages of hypocalcemia between two different CS volume groups (≤ 20 cc group and > 20 cc groups) (Table 2) at any time points. However, no patient suffered from hypercalcemia in this cohort, at any time points.

### 3.4. Changes of Serum Calcium Levels by Time and Differences of Serum Calcium Levels by CS Volume

**Figure 2:**
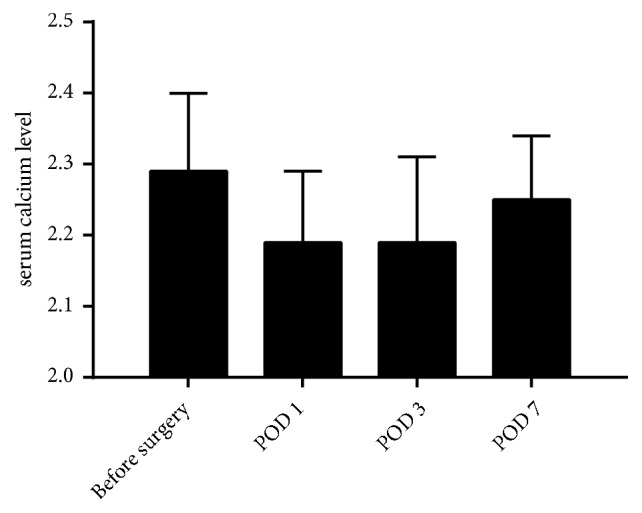
Changes of serum calcium levels before and after CS implantation surgery.

Significant difference was identified regarding serum calcium levels among different time points (*P *< 0.001). Outcomes of post hoc multiple comparisons revealed that, compared with preoperative level (2.29 ± 0.11 mmol/L), serum calcium levels significantly decreased on the 1st (2.19 ± 0.10 mmol/L) (*P* < 0.001) and 3rd PODs (*P *< 0.001) (2.19 ± 0.12 mmol/L) and back to preoperative level on the 7th POD (2.25 ± 0.09 mmol/L) (*P *= 0.334) (Figure 2). As revealed in Table 2, no statistical differences were found regarding serum calcium levels between the two CS volume groups at any time points (*P *> 0.05).

### 3.5. Potential Links between CS Volume and Postoperative Serum Calcium Levels and Potential Associations between Preoperative Serum Calcium Level and Postoperative Calcium Levels

Outcomes showed that no significant links were found between CS volume and postoperative serum calcium levels on the 1st (*P* = 0.521), 3rd (*P* = 0.681), or the 7th PODs (*P* = 0.214). However, statistical associations were observed between preoperative calcium level and postoperative calcium levels on the 3rd (*P* = 0.019) and 7th PODs (*P* = 0.036). There was no statistical association between preoperative calcium level and calcium level on the 1st POD (*P* = 0.308)

## 4. Discussion

Hypercalcemia, a frequent disorder with prevalence in the general population of approximate 1/1000, mostly results from primary hyperparathyroidism or malignancy though there exist many other causes [14]. The most intuitive presentation of hypercalcemia is detection of an elevated level of serum calcium. Outcomes of the present study demonstrated that, quite different from what had occurred in PJI patients, in this cohort, no patient had developed hypercalcemia before and after CS implantation. On the contrary, prevalence of asymptomatic hypocalcemia was more frequent in patients with posttraumatic OM. Based on the outcomes, we concluded that hypercalcemia may not be a frequent complication after local CS use during the treatment of extremity posttraumatic OM.

Recently, as an antibiotic delivery vehicle, CS is increasingly used for the treatment of osteoarticular infections and has obtained satisfying efficacy. Ferguson et al. [8] reviewed efficacy of different CS products for the treatment of OM [4, 5, 15–21]. Outcomes revealed that infection clearance ranged from 80% [17, 21] to 100% [16], with an average of 90.37% (366 out of 405 patients). In addition, Morley et al. [6] mixed CS with gentamicin and vancomycin for the treatment of a deep DF infection patient and also achieved a fine outcome. Similarly, Jogia et al. [22] reported use of CS with gentamicin and vancomycin for the management of DF OM in 20 patients, with no infection recurrence or adverse reactions, which provides a new strategy for DF therapy. Moreover, Kumar et al. [23] used CS as a bone graft substitute for the treatment of osseous bone defects following curettage of benign lesions, OM, and trauma, with good efficacy and without significant complications.

The aforementioned satisfying efficacy of CS for the treatment of osteoarticular infections and bone defects never means it has no flaws. Firstly, CS is less strong than PMMA and cannot provide structural support for stabilization of bony structure [7, 24]. Secondly, aseptic wound leak is one of the most frequent complications following CS local use [8], which ranged from 6% [4] to 33.3% [19], with an average of 16.3% (62 out of 380 patients). Thirdly, the fracture risk after CS local use as a bone void filler was from 0% [16, 20] to 14% [18], with an average of 4.7% (17 out of 365 patients). Fourthly, apart from the frequently occurring complications, there are still rare adverse effects, such as HO. Previous study had indicated the HO rate following local use of CS was 1.2% [9], which probably associates with CS volume implanted during surgery. Meanwhile in a recent study [9] Kallala and Haddad reported a rare complication of hypercalcemia following CS local implantation as an antibiotic carrier during PJI therapy, which occurred in 20% patients. Currently, hypercalcemia following CS use has been seldom reported [23]. Outcomes of the study have aroused wide concerns regarding its safety.

In the present study, we monitored serum calcium levels before and after CS implantation for the treatment of posttraumatic OM and found that no patient experienced hypercalcemia before and within 7 days after surgery. On the contrary, hypocalcemia was more frequent, with a prevalence of 16.4% preoperatively. We are still unclear about potential relationship between hypocalcemia and posttraumatic OM. That is to say, whether hypocalcemia increases the susceptibility to OM or long term bone infection results in elevated risk of hypocalcemia remains unclear. Therefore, potential mechanisms should be further explored. Prevalence of hypocalcemia sharply increased on the 1st and 3rd PODs and decreased close to preoperative rate on the 7th POD. These results suggested that surgical intervention may influence serum calcium level. Despite the high rates of hypocalcemia among OM patients before and after surgery, no patient experienced clinical symptoms of hypocalcemia.

In addition to the above findings, we also observed that CS volume used during surgery did not affect postoperative serum calcium levels. According to a previous study [9], we divided patients into a larger CS volume group and a smaller CS volume group by 20 cc. Outcomes revealed that no significant differences were found regarding the prevalence of hypocalcemia or serum calcium levels at any time points postoperatively. Additionally, no statistical links were found between intraoperative CS volume and postoperative serum calcium levels at any time points. However, significant associations were observed between preoperative serum calcium level and those on the 3rd and 7th PODs. These outcomes imply that postoperative serum calcium levels may be affected by preoperative level and surgical interventions but not local CS implantation.

As each local antibiotic delivery vehicle has advantages and disadvantages, recently, many material science investigations have been performed to maximize antibacterial effects and minimize adverse effects of different antibiotic carriers. Cyphert et al. [25] reported that an additive of insoluble cyclodextrin microparticles into traditional PMMA cement promotes postimplantation antibiotic refilling, broadens range of compatible antibiotics, and prolongs antimicrobial therapy while preserving its favorable mechanical properties. Additionally, Mi et al. [26] summarized current experiences towards the use of nanoparticles and nanostructured surfaces to combat bacterial growth and infections, which is a promising solution to the growing problem of antibiotic resistance and biofilm-related device infections.

The current study has several limitations. Firstly, the number of the included participants was still limited. Future studies with a larger sample size are warranted to obtain more accurate outcomes. Secondly, we monitored serum calcium levels for only 7 days after surgery in most cases, which is not enough. To better evaluate potential effects of CS use on serum calcium level, a longer follow-up time (until the complete degradation of CS) may be necessary. Thirdly we used total calcium as the indicator to diagnose hypercalcemia or hypocalcemia, where biases may exist. Consequently, cautious attitude should be taken towards the outcomes. Lastly, this study only reported potential risks following CS use in patients with posttraumatic OM; whether such risk may occur in other types of COM (e.g., chronic hematogenous and DF OM) remains unclear. Therefore, potential risk of CS application in other types of COM should be further investigated.

## 5. Conclusions

In summary, outcomes of the present study suggested that, in this Chinese cohort with posttraumatic OM, asymptomatic hypocalcemia appeared to be more frequent. Local CS use may not affect serum calcium level, the latter of which may be influenced by preoperative calcium level and surgical interventions.

## Figures and Tables

**Table 1:** Clinical characteristics of the included patients with extremity posttraumatic OM.

Clinical characteristics	Outcomes
Infection site distribution (left/right/bilateral)	22/31/2
Infection site no. distribution (single/multiple)	51/4
Single infection site distribution (no., %)	
Tibia	32 (62.74%)
Calcaneus	8 (15.69%)
Femur	7 (13.72%)
Humerus	1 (1.96%)
Radius	1 (1.96%)
Index finger	1 (1.96%)
The 5th metatarsal	1 (1.96%)
Positive rate of pathogen culture (%, E/T)	57.78% (26/45)
Pathogen for infection (monomicrobial/polymicrobial)	24/2
Pathogen for monomicrobial infection (no., %)	
*Staphylococcus aureus*	6 (25%)
*Pseudomonas aeruginosa*	3 (12.5%)
*Enterococcus faecalis*	3 (12.5%)
Other pathogens *∗*	12 (50%)

*∗* Other pathogens included *Acinetobacter baumannii* 1 case, *Klebsiella* 1 case, *Enterococcus avium* 1 case, *Proteus penneri* 1 case, *Proteus mirabilis* 1 case, *Gemella haemolysans* 1 case, *Staphylococcus haemolyticus* 1 case, *Enterococcus faecium* 1 case, *Providencia* 1 case, *Micrococcus luteus* 1 case, *Achromobacter* 1 case, and *Enterobacter cloacae* 1 case.

**Table 2:** Prevalence of hypocalcaemia and serum calcium levels between different CS volume groups at each time point.

Time points	Prevalence of hypocalcaemia			Serum calcium levels		
	≤20 cc group	>20 cc group	*P *value	≤ 20 cc group	>20 cc group	*P *value
	% (E/T)	% (E/T)		(M ± SD)	(M ± SD)	
Pre-surgery	19.4 (7/36)	10.5 (2/19)	0.641	2.30 ± 0.12	2.28 ± 0.07	0.481
1st POD	61.1 (22/36)	57.9 (11/19)	0.817	2.19 ± 0.10	2.20 ± 0.11	0.679
3rd POD	54.3 (19/35)	52.9 (9/17)	0.927	2.21 ± 0.13	2.17 ± 0.11	0.311
7th POD	29.4 (5/17)	20 (3/15)	0.691	2.24 ± 0.08	2.26 ± 0.10	0.512

CS: calcium sulfate; E/T: events/total; M: mean; SD: standard deviation; POD: postoperative day.

## Data Availability

The datasets of the present study are available from the corresponding authors on reasonable request.

## Abbreviations

CS: Calcium sulfate
PJI: Periprosthetic joint infection
OM: Osteomyelitis
POD: Postoperative day
COM: Chronic osteomyelitis
PMMA: Polymethylmethacrylate
HO: Heterotopic ossification
DF: Diabetic foot
RIA: Reamer-Irrigator-Aspirator
SPSS: Statistical Product and Service Solutions
IQR: Interquartile range
ANOVA: One-way analysis of variance
M: Mean
SD: Standard deviation.
